# DNA sequence-based re-assessment of archived *Cronobacter sakazakii* strains isolated from dairy products imported into China between 2005 and 2006

**DOI:** 10.1186/s12864-018-4881-9

**Published:** 2018-06-28

**Authors:** Qingyan Guo, Jielin Yang, S. J. Forsythe, Yuan Jiang, Wei Han, Yuping He, Bing Niu

**Affiliations:** 1 Shanghai Customs (former Shanghai Entry-Exit Inspection and Quarantine Bureau), 1208 Minsheng Road, Shanghai, 200135 People’s Republic of China; 2 Technical Center for Animal, Plant and Food Inspection and Quarantine, Shanghai Entry-Exit Inspection and Quarantine Bureau, 1208 Minsheng Road, Shanghai, 200135 People’s Republic of China; 3 foodmicrobe.com, Adams Hill, Keyworth, Nottinghamshire, NG12 5GY UK; 4 Shanghai University, 99 Shangda Road, Shanghai, 200135 People’s Republic of China

**Keywords:** *Cronobacter sakazakii*, MLST, WGS, SNP analysis, Dairy products, Traceability

## Abstract

**Background:**

*Cronobacter* species are associated with severe foodborne infections in neonates and infants, with particular pathovars associated with specific clinical presentations. However, before 2008 the genus was regarded as a single species named *Enterobacter sakazakii* which was subdivided into 8 phenotypes. This study re-analyzed, using multi-locus sequence typing (MLST) and whole genome sequence with single nucleotide polymorphism analysis (WGS-SNP), 52 strains which had been identified as *Enterobacter sakazakii* as according to the convention at the time of isolation. These strains had been isolated from dairy product imports into China from 9 countries between 2005 and 6. Bioinformatic analysis was then used to analyze the relatedness and global dissemination of these strains.

**Result:**

*FusA* allele sequencing revealed that 49/52 strains were *Cronobacter sakazakii,* while the remaining 3 strains were *Escherichia coli, Enterobacter cloacae,* and *Franconibacter helveticus*. The *C. sakazakii* strains comprised of 8 sequence types (STs) which included the neonatal pathovars ST1, ST4 and ST12. The predominant sequence type was ST13 (65.3%, 32/49) which had been isolated from dairy products imported from 6 countries. WGS-SNP analysis of the 32 *C. sakazakii* ST13 strains revealed 5 clusters and 5 unique strains which did not correlate with the country of product origin.

**Conclusion:**

The mis-identification of *E. coli, E. cloacae* and *F. helveticus* as *Cronobacter* spp. reinforces the need to apply reliable methods to reduce the incidence of false positive and false negative results which may be of clinical significance. The WGS-SNP analysis demonstrated that indistinguishable *Cronobacter* strains within a sequence type can be unrelated, and may originate from multiple sources. The use of WGS-SNP analysis to distinguishing of strains within a sequence type has important relevance for tracing the source of outbreaks due to *Cronobacter* spp.

**Electronic supplementary material:**

The online version of this article (10.1186/s12864-018-4881-9) contains supplementary material, which is available to authorized users.

## Background

Until 2007 the *Cronobacter* genus was known as the single species *Enterobacter sakazakii*. The organism is a member of the Enterobacteriaceae, and are facultative anaerobic, oxidase-negative, catalase-positive, rod-shaped, motile, non-spore-formers [1]. About 80% of strains produce a yellow pigment at room temperature [1]. The *Cronobacter* genus is composed of seven species, namely *C. sakazakii*, *C. malonaticus*, *C. turicensis*, *C. muytjensii*, *C. dublinensis*, *C. universalis*, and *C. condimenti* [1, 2]. However, *C. sakazakii* and *C. malonaticus* are particularly associated with infant and adult infections, respectively [3–5]. Most *Cronobacter* infections are in the adult population and are non-life threatening; however, there is a high fatality rate (40–80%) and permanent neurological sequelae following *Cronobacter* infections of pre-term, low-birth-weight neonates and infants [6, 7]. *C. sakazakii* is the species of most concern in neonatal and infant infections as it is linked to cases of meningitis, sepsis and necrotizing enterocolitis. Reconstituted powdered infant formula (PIF) and contaminated expressed breast milk have been implicated as the sources of *Cronobacter* infection in a number of neonatal and infant cases [4]. According to Minor et al. (2015), *Cronobacter* is the most expensive food-associated infection due to the loss of life and debilitation of survivors, each case being estimated to cost $1 million USD [8].

*Cronobacter* species are ubiquitous and have been isolated from a variety of foods including PIF, dairy products, dairy product ingredients, ready-to-eat foods and various environmental sources including hospital air, water and equipment used for PIF reconstitution, and milk powder manufacturing plants [9–12]. The organism can survive for more than two years in a desiccated environment such as PIF [13].

In 2002, the International Commission on Microbiological Specifications for Foods (ICMSF) designated *Cronobacter,* then known as *E. sakazakii,* as a severe hazard for restricted populations of immunocompromised individuals such as neonates and infants [14, 15]. Since the organism is foodborne, the Food and Drug Administration (FDA) in China has proposed that the organism should be absent in infant feeds.

Since the mid-1990s, China has been a net-importer of dairy products, and this increased in 2004 following domestic dairy-safety incidents. Dairy products in China are mainly imported from New Zealand, Australia, France, Germany, and the United States. In these countries a number of clinical outbreaks of neonatal infections have been reported which were attributed to *Cronobacter* contaminated reconstituted PIF [6, 7, 16–18]. It is therefore plausible that bacterially contaminated PIF was exported from these countries. However, to date, no data has been collated on *Cronobacter* spp. isolated from dairy products imported into China. This would be useful for microbial source tracking of *Cronobacter* through contaminated dairy products, and subsequently reduce future risk of neonatal and infant infections.

Previously, pulsed-field gel electrophoresis (PFGE) was regarded as a gold-standard method for molecular-epidemiological investigations of the outbreaks of pathogens [19]. However, PFGE analysis has several limitations including being unable to distinguish between unrelated strains of highly clonal organisms such as *Cronobacter* spp. [20]. Due to such issues, FoodNet is transitioning away from PFGE to WGS-SNP for its epidemiological analysis of foodborne outbreaks [21, 22].

Whole genome comparisons reveal a plethora of genetic polymorphisms that can be used for fast and accurate discrimination of infection-associated isolates; even down to the single strain level. WGS offers a higherresolution of the genetic relatedness of isolates than did PFGE and provides additional information regarding multi-locus sequence type (MLST), CRISPR-*cas* arrays, single nucleotide polymorphisms (SNPs), pathogenicity genes, and detailed molecular characterization of strains [1, 23].

There are several examples of comparison studies between conventional bacterial genotyping and WGS in the literature. Lytsy et al. (2017) compared the reliability, resolution, and applicability of PFGE, WGS and MLST for vancomycin-resistant *enterococci* (VRE), leading to the recommend of WGS-ANI analysis for epidemiological issues of VRE [24]. McRobb et al. (2015) combined whole-genome indel analysis and SNP phylogeny for *Burkholderia pseudomallei,* which provided greater resolution and a better fit with the epidemiological chronology of events [25]. Ferdous et al. (2016) undertook WGS analysis of 132 shiga toxin-producing *E. coli* (STEC) isolates [26]. These isolates constituted 44 sequence types (STs), 42 serogenotypes and 14 stx subtype combinations. Such studies demonstrate the application of WGS to the microbial source tracking and risk assessment. This comparative WGS study of *C. sakazakii* isolates from dairy products collected over 2 years is expected to be similarly informative and useful.

The aim of this work was to re-identify and genotype, using 7-loci MLST and WGS, bacterial isolates from imported dairy products which had previously been identified as *E. sakazakii*. SNP analysis was then used to distinguish between strains within the predominant sequence type, *C. sakazakii* ST13, which had been originally isolated from various dairy products originating from six countries.

## Method

### Dairy product sample collection

The origin of the isolates used in this study is given in Table 1. These isolates had been obtained from imported dairy products which had been sampled at the Shanghai Entry-Exit Inspection and Quarantine Bureau between 2005 and 2006. A total of 52 imported dairy products, including PIF, raw dairy product ingredients, cheeses, milk powder and other dairy items collected were found to be contaminated by the organism then known as *E. sakazakii*. For the purposes of understanding discrepancies in the identification of these strains, the original isolation and identification methods used by previous workers are described below.

**Table 1:** Detail information of all isolates, Clustering of *Cronobacter* sequence type by MLST and O-serotypes according to *gnd* and *gal*F sequence analysis

Isolate	PubMLST ID number	API20E bionumber	Organism	ST	O-serotype^a^	*galF* loci	*gnd* loci	Source	Country	Year
CS-6	1956	06257300153722011	*C. sakazakii*	13	O:2	25	28	Powdered infant formula	New Zealand	2005
CS-8	1957	06257300153722011	*C. sakazakii*	13	O:2	25	28	Powdered infant formula	New Zealand	2005
CS-25	1967	06257300153722011	*C. sakazakii*	13	O:2	25	28	Powdered infant formula	New Zealand	2005
CS-30	1968	06257300153722011	*C. sakazakii*	13	O:2	25	28	Powdered infant formula	New Zealand	2005
CS-36	1972	06257300153722011	*C. sakazakii*	13	O:2	25	28	Powdered infant formula	New Zealand	2006
CS-45	1978	06257300153722011	*C. sakazakii*	13	O:2	25	28	Powdered infant formula	New Zealand	2006
CS-48	1980	06257300153722011	*C. sakazakii*	13	O:2	25	28	Powdered infant formula	New Zealand	2006
CS-52	1983	06257300153722011	*C. sakazakii*	13	O:2	25	28	Powdered infant formula	New Zealand	2006
CS-72	2000	06257300153722011	*C. sakazakii*	13	O:2	25	28	Powdered infant formula	New Zealand	2006
CS-2	1953	06257300153722011	*C. sakazakii*	13	O:2	25	28	Powdered infant formula	Netherlands	2005
CS-1	1952	06257300153722011	*C. sakazakii*	13	O:2	25	28	Dairy product ingredient	USA	2005
CS-62	1992	06257300153722011	*C. sakazakii*	13	O:2	25	28	Dairy product ingredient	USA	2006
CS-65	1995	06257300153722011	*C. sakazakii*	13	O:2	25	28	Dairy product ingredient	USA	2006
CS-66	1996	06257300153722011	*C. sakazakii*	13	O:2	25	28	Dairy product ingredient	USA	2006
CS-9	1958	06257300153722011	*C. sakazakii*	13	O:2	25	28	Dairy product ingredient	New Zealand	2005
CS-17	1963	06257300153722011	*C. sakazakii*	13	O:2	25	28	Dairy product ingredient	Australia	2005
CS-47	1979	06257300153722011	*C. sakazakii*	13	O:2	25	28	Dairy product ingredient	France	2006
CS-64	1994	06257300153722011	*C. sakazakii*	13	O:2	25	28	Dairy product ingredient	Belgium	2006
CS-18	1964	06257300153722011	*C. sakazakii*	13	O:2	25	28	Milk powder	New Zealand	2005
CS-19	1965	06257300153722011	*C. sakazakii*	13	O:2	25	28	Milk powder	New Zealand	2005
CS-61	1991	06257300153722011	*C. sakazakii*	13	O:2	25	28	Milk powder	New Zealand	2006
CS-56	1987	06257300153722011	*C. sakazakii*	13	O:2	25	28	Milk powder	New Zealand	2006
CS-60	1990	06257300153722011	*C. sakazakii*	13	O:2	25	28	Milk powder	New Zealand	2006
CS-14	1961	06257300153722011	*C. sakazakii*	13	O:2	25	28	Milk powder	USA	2005
CS-71	1999	06257300153722011	*C. sakazakii*	13	O:2	25	28	Milk powder	France	2006
CS-34	1971	06257300153722011	*C. sakazakii*	13	O:2	25	28	Cheese	Australia	2006
CS-37	1973	06257300153722011	*C. sakazakii*	13	O:2	25	28	Cheese	Australia	2006
CS-38	1974	06257300153722011	*C. sakazakii*	13	O:2	25	28	Cheese	Australia	2006
CS-33	1970	06257300153722011	*C. sakazakii*	13	O:2	25	28	Cheese	France	2006
CS-44	1977	06257300153722011	*C. sakazakii*	13	O:2	25	28	Cheese	France	2006
CS-32	1969	06257300153722011	*C. sakazakii*	13	O:2	25	28	Other dairy products	New Zealand	2005
CS-63	1993	06257300153722011	*C. sakazakii*	13	O:2	25	28	Other dairy products	USA	2006
CS-13	1960	06257300153722011	*C. sakazakii*	4	O:2	3	2	Dairy product ingredient	USA	2005
CS-54	1985	06257300153722011	*C. sakazakii*	4	na	4	45/46	Dairy product ingredient	USA	2006
CS-15	1962	06257300153722011	*C. sakazakii*	4	O:2	3	2	Other dairy products	USA	2005
CS-58	1989	06257300153722011	*C. sakazakii*	4	na	4	45/46	Other dairy products	New Zealand	2006
CS-70	1998	06257300153722011	*C. sakazakii*	4	O:2	3	2	Milk powder	New Zealand	2006
CS-43	1976	06257300153722011	*C. sakazakii*	4	O:2	3	2	Cheese	Italy	2006
CS-55	1986	06257300153722011	*C. sakazakii*	375	na	26	67	Dairy product ingredient	New Zealand	2006
CS-53	1984	06257300153722011	*C. sakazakii*	375	na	26	67	Other dairy products	New Zealand	2006
CS-5	1955	06257300153722011	*C. sakazakii*	375	na	26	67	Dairy product ingredient	Poland	2005
CS-10	1959	06257300153722011	*C. sakazakii*	375	na	26	67	Other dairy products	Poland	2005
CS-49	1981	06257300153722011	*C. sakazakii*	1	O:1	2	1	Whole milk powder	New Zealand	2006
CS-24	1966	06257300153722011	*C. sakazakii*	1	O:1	2	1	Other dairy products	New Zealand	2005
CS-67	1997	06257300153722011	*C. sakazakii*	21	O:1	19	21	Dairy product ingredient	Canada	2006
CS-42	1975	06257300153722011	*C. sakazakii*	21	O:1	19	21	Other dairy products	Canada	2006
CS-51	1982	06257300153722011	*C. sakazakii*	12	na	26	62	Other dairy products	New Zealand	2006
CS-4	1954	06257300153722011	*C. sakazakii*	42	na	/	/	Dairy product ingredient	Poland	2005
CS-57	1988	06257300153722011	*C. sakazakii*	233	na	47	47	Other dairy products	New Zealand	2006
CS-22	2449	2,405,610,540,464,611	*E. coli*	na	na	na	na	Dairy product ingredient	France	2005
CS-35	2451	0627634553532210	*E. cloacae*	na	na	na	na	Powdered infant formula	Netherlands	2006
CS-23	2450	0605710142500210	*F. helveticus*	na	na	na	na	Powdered infant formula	New Zealand	2005

^a^The O-serotype was predicted using the whole genome sequence by comparison with the defined *C. sakazakii* O-serotype regions in *Cronobacter* database [34]

^b^*na* not applicable

### Original *E. sakazakii* isolation procedures

The dairy products had been analyzed as according to the then recommended methods of the US FDA and Health Canada’s Department of Health Products and Food [27–30]. Samples (40 g) were placed in an Erlenmeyer flask containing 360 ml of sterilized water which had been preheated to 45 °C and gently shaken to dissolve well before incubation overnight at 36 °C. The mixture (10 ml) was then pipetted into 90 ml sterile Enterobacteriaceae enrichment broth and incubated at 36 °C. After overnight incubation, the enrichment broth was streaked on crystal violet neutral red bile salt glucose agar plates and incubated at 36 °C overnight. Any resulting purple or pink colonies were streaked for single colony isolation on tryptic soy agar (TSA) plates which were incubated at 25 °C for up to 72 h. Colonies from the TSA agar plates were picked, and identified using API 20E phenotyping kits as well as the oxidase test. These strains were then archived at − 80 °C using magnetic beads until recovery on TSA for this study.

### Genome sequence

Genomic DNA of the bacteria was extracted by the cetyl trimethyl ammonium bromide(CTAB) method and sent to the Beijing Novogene Bioinformatics Technology Co. Ltd. for processing. The harvested DNA was quantified by Qubit before WGS was performed using the Illumina HiSeq 2500-PE150 platform with massively parallel sequencing (MPS) Illumina technology. The sequences were A-tailed, ligated to paired-end adaptors and PCR amplified with a 350 bp insert and a mate-pair library with an insert size of 6 kb for library construction.

### Genome assembly

Illumina PCR-adapter reads and low-quality reads from the paired-end and mate-pair libraries were filtered by the quality control step of the coupling pipeline. All good-quality paired reads were assembled into scaffolds using SOAP de novo (version 2.04) software [31, 32]. The filtered reads were then used for gap closing and genome assembly. And the sequences have been deposited in the PubMLST database under The PubMLST ID number listed in Table 1.

### MLST analysis

*Cronobacter* 7-loci MLST profiling was performed by submitting the FASTA genome sequences to the curator (SJF) of the open access *Cronobacter* PubMLST database (https://pubmlst.org/*cronobacter*/). The 7-loci being ATP synthase b chain (*atpD*), elongation factor G (*fusA*), glutaminyl tRNA synthetase (*glnS*), glutamate synthase large subunit (*gltB*), DNA gyrase subunit B (*gyrB)*, translation initiation factor IF-2 (*infB*) and phosphoenolpyruvate synthase A (*ppsA*). Speciation of *Cronobacter* strains was achieved by phylogenetic analysis of the *fusA* allele sequence [1, 5, 33]. The sequence types (ST) and clonal complexes (CC) for the isolates were predicted using the BIGSdb tools within the database [5].

### O-serotype, *galF* and *gnd* loci determination analysis

The *galF* and *gnd* loci, flanking genes for the O-serotype region, were assigned from the uploaded genome sequences using the BIGSdb tools within the PubMLST database [34]. The O-antigen was then predicted from the *galF* and *gnd* loci as according to Ogrodzki & Forsythe [34].

### Phylogenetic analyses of *C. sakazakii* ST13 strains based on SNP differences

WGS was undertaken for SNP analysis of the 32 strains belonged to *C. sakazakii* ST13 using SMALT and SAMtools [35, 36]. The SNP calling was done independently for each cluster using the earliest isolate within the cluster as the index strain. The resulting variant call format(VCF) files were filtered using VCFTools to include only SNPs with minimum quality score of 30, minimum depth of 8.0, and minimum allele frequency of 0.90 [37–39]. SNPs in each cluster were concatenated and used to create a maximum likelihood phylogeny by using RAxML with the GTR-gamma model [40]. The resulting trees were visualised and annotated using Figtree (http://tree.bio.ed.ac.uk/software/figtree/).

## Results

### Re-identification of archived *E. sakazakii* strains

Fifty-two bacterial strains which had been isolated in 2005–2006 from imported dairy products and identified using phenotyping as *E. sakazakii* were re-identified using *fusA* allele sequencing. The majority (49/52) of strains were identified as *C. sakazakii* (Additional file 1: Figure S1)*.* The remaining three strains (CS-22, CS-23, & CS-35) were identified as *Escherichia coli*, *Franconibacter helveticus*, and *Enterobacter cloacae* respectively.

### MLST analysis of *Cronobacter* isolates

The MLST results for the 49 *C. sakazakii* isolates are summarized in Table 1. There was relatively low diversity across the *C. sakazakii* strains. The majority (32/49) of strains were in sequence type 13 (ST13), with the remaining strains in ST4 (6), ST375 (4), ST1 (2), ST21 (2), ST12 (1), ST42 (1), and ST233 (1). Ten of the ST13 strains had been isolated from PIF. The remaining strainsr were from dairy product ingredients (8), milk powder (7), cheese (5), and other dairy products (2). The six *C. sakazakii* ST4 strains (pathovar associated with neonatal meningitis) were from milk powder, cheese, other dairy products and dairy product ingredients; Table 1. None were from PIF. They had been imported from USA, New Zealand, and Italy. Four (8.1%) isolates belong to ST375 and had been isolated from dairy products and dairy product ingredients. The remaining *C. sakazakii* strains had been isolated from dairy product ingredients and dairy products other than PIF, milk powder or cheese.

The strains of *E. coli, E. cloacae* and *F. helveticus* which had been mis-identified as *C. sakazakii,* had been isolated from PIF and dairy product ingredients.

### Predicted O-serotype

The O-serotype was predicted based on the DNA sequence of the flanking genes *galF* and *gnd,* as according to the in silico scheme of Ogrodzki and Forsythe (2015) [31]. The 32 ST13 strains were all serotype Csak O:2, *galF* 25 and *gnd* 28. They were therefore indistinguishable; Table 1. The Csak O:2 serotype was not unique to ST13 as it was also found in the four ST4 strains with the corresponding loci *galF*3 and *gnd*2. No serotype could be assigned to ST4 strains with loci *galF* 4 and *gnd* 45, as no O-antigen genes has been reported to date with these flanking loci. Similarly, no serotype could be allocated to the 7 strains in ST375 (4), ST12 (1), ST42 (1) or ST233 (1). Only two *C. sakazakii* O-serotypes were found (Csak O:1 & O:2), whereas there were 8 loci variants of *galF* and *gnd.*

### WGS-SNP analysis of *C. sakazakii* belonging to ST13

Due to the highly level of clonality with the *C. sakazakii* species*,* 7-loci MLST is unable to distinguish between unrelated strains within the same sequence type [1, 5]. It was considered highly probably that some of the 32 ST13 strains were unrelated since they had been originally isolated from different dairy products imported from various countries; Australia, New Zealand, France, USA, Belgium and Netherlands. Therefore the 32 ST13 isolates were whole genome sequenced for further genotyping and SNP analysis. The earliest isolate of this cluster was *C. sakazakii* CS-1 isolated from dairy product ingredients imported from USA in 2005. This strain was used as the index strain for SNP calls in order to determine strain relatedness. The SNP differences for each of the strains was determined using SAMTools and the SNP phylogeny was constructed (Fig. 2).

The SNP tree topology showed the 32 strains formed five clusters and five single isolates. There was no pattern with respect to country of origin, or dairy product with any of the clusters. The SNP differences were mainly < 60, except for CS71 which had 5387 SNPs. The five strains in cluster 1 had a high degree of similarity with the index strain as shown by the small number of SNPs different; a maximum of 11 SNP. Cluster 5 was more diffuse than the other 4 clusters I had the largest number of strains (12) with between 4 and 11 SNP differences to the index strain CS1.

## Discussion

MLST is regarded as an unambiguous and expansive method for the differentiation of strains in the *Cronobacter* genus, with the number of loci varying between 7 for conventional MLST, 51 with ribosomal-MLST, and 1836 with core-genome MLST [1, 5]. It is considerably more reliable than phenotyping using commercial kits which is reported to have an accuracy of between 43.2–88.9% for members of the *Cronobacter* genus [41]. The *fusA* allele is congruent with the phylogeny of the genus and can be used for speciation [33]. The remaining 6 alleles can then be determined for 7-loci MLST profiling. The 52 strains previously identified as *E. sakazakii* were re-identified using *fusA* allele sequence phylogeny. This revealed that the original isolation and phenotyping-based detection method had mis-identified three strains of *E. coli*, *E. cloacae* and *F. helveticus* as *E. sakazakii.* Such mis-identifications may have resulted in product rejection at port of entry due to the false positive result. This reinforces the need to apply DNA-sequence based methods rather than out of date phenotyping methods for identifying presumptive *Cronobacter* isolates, especially when regulatory action may be taken.

**Fig. 1:**
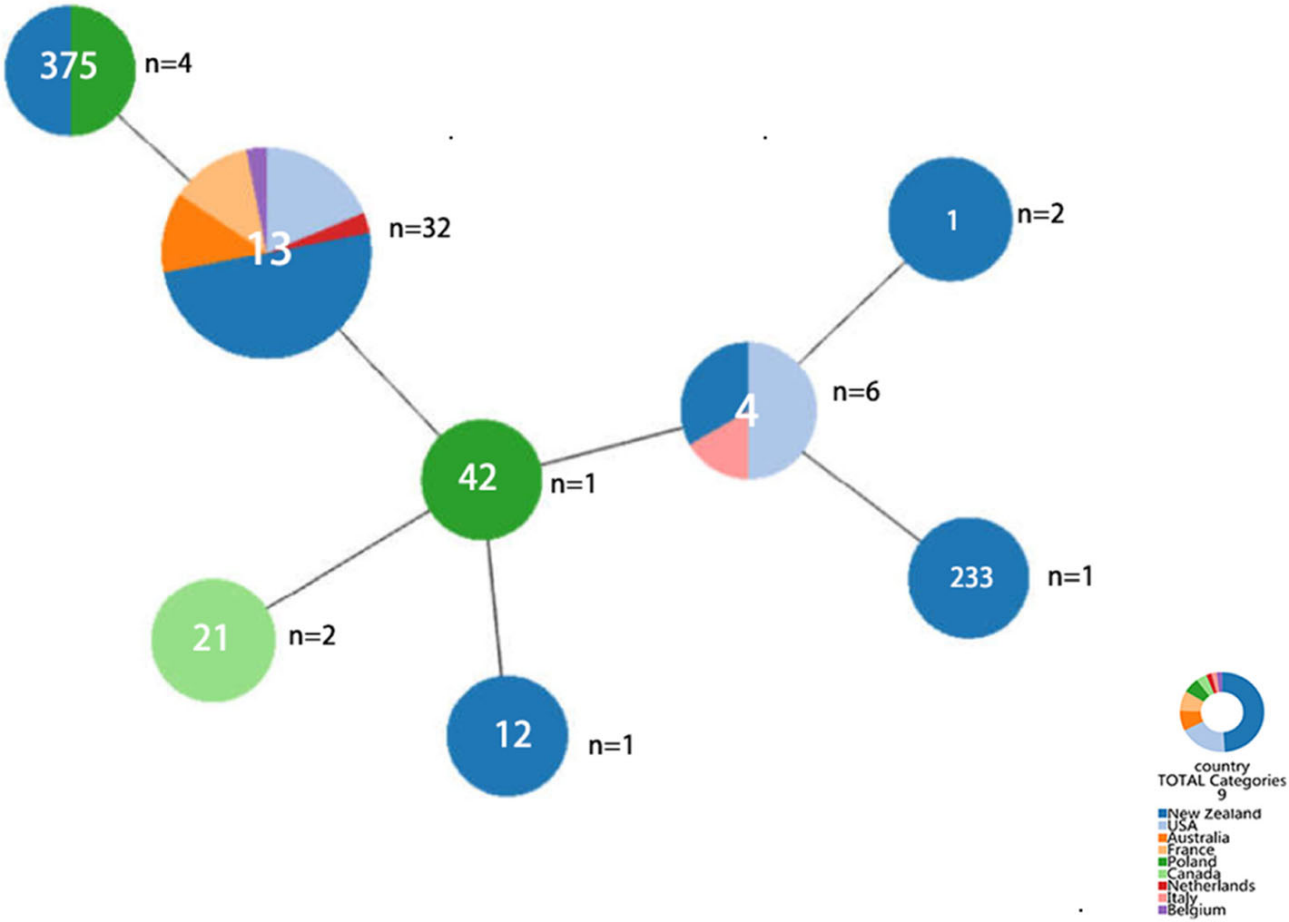
Population diversity of the C. sakazakii strains from dairy products accordingto sequence type and country of origin. Figure was generated using the GoeBURSTalgorithm. The threshold for the output was set to triple locus variation. "*n=1*" represents the number of isolates in the pie chart. The dominant STs are representedby the circles with larger diameters

The remaining 49 isolates were all strains of *C. sakazakii.* These strains were in 8 sequence types, three of which were represented by only a single isolate. These strains had originated from Oceania, Europe and North America (Fig. 1). The most frequently occurring sequence type was *C. sakazakii* ST13 (66%, 32/49). The ST13 strains were from various dairy products imported from six countries, though half (16/32) were from New Zealand. Ten strains had been isolated from PIF, and these had primarily (9/10) been imported from New Zealand. In 1994, 26 isolates of *C. sakazakii* were obtained from an outbreak at a neonatal intensive care unit (NICU) in France that lasted over 3 months and claimed the lives of three neonates; 5 of the 26 isolates were ST13 [6, 18]. In addition, PIF was linked to a neonatal death due to *C. sakazakii* ST4 infection in New Zealand in 2004, which was close to the sampling period of this study [42]. Therefore, the presence of *C. sakazakii* ST13 in the PIF imported into China indicates that there was some risk of infection to infants in 2005–2006. Nevertheless, there were no reported cases of *Cronobacte*r infections in neonates or infants in China until 2014 [43].

*C. sakazakii* ST4 was the second most prominent ST isolated (12%, 6/49). This sequence type is highly significant as it is the pathovar which is strongly associated with neonatal meningitis [1, 44, 45]. Although none of the isolates had been isolated from PIF, the six ST4 strains had been isolated from PIF ingredients (whey powder and whey protein powder), as well as cheese, milk powder and other dairy products. These had been imported from USA, Italy, and New Zealand, reflecting the wide distribution of this sequence type in food products.

Two isolates (CS-24, CS-49), isolated from milk powder and other dairy goods imported from New Zealand were *C. sakazakii* ST1. This sequence type is associated with clinical infections and some cases of neonatal meningitis [1, 44, 45]. There was one ST12 *C. sakazakii* isolate from dairy products from New Zealand. This is the *C. sakazakii* pathovar associated with necrotizing enterocolitis in neonates [1, 5, 7, 44].

Four strains of ST375 (8%, *n* = 4) had been isolated between 2005 and 2006. These belong to clonal complex 73 (CC73), being a single locus SNP variant of ST73. The PubMLST database only has one other record of *C. sakazakii* ST73 isolation in China, which from imported food in 2009. The remaining strains in this study comprised of single strains of ST42 and ST233 which had been isolated from imports from Poland and New Zealand.

At the national level, more than half of the *C. sakazakii* isolates (24/49) were from New Zealand. These isolates were heterogenous, belonging to six sequence types, of which ST13 was the most abundant at 66.7% (Fig. 1). However, the *Cronobacter* PubMLST database, only contains the records of three *C. sakazakii* isolates from New Zealand other than those reported here, and none were ST13. However, PIF was linked to a fatal neonatal infection by *C. sakazakii* ST4 in New Zealand in 2004, 2 years before the sampling period of this study [38]. The strains from the USA consisted of only ST13 and ST4. Compared with other countries, the USA had the most *C. sakazakii* meningitic ST4 pathovar isolates (3/9). Meanwhile, France, Australia, the Netherlands, and Belgium have only one sequence type; ST13.

In terms of product categories, *C. sakazakii* ST13 was recovered from five types of dairy products. It is notable that all strains isolated from PIF belonged to ST13. Dairy product ingredients containing *C. sakazakii* ST13 included whey powder and whey protein powder, which are used for the production of PIF. Dairy product ingredients had a range of *C. sakazakii* sequence types, including ST13 and ST4, which indicates that imported dairy product ingredients are still a risk to infant health. Six isolates were from cheese, of which one was ST4 and the remaining five belonged to ST13. This indicates that cheese, as a food which may be consumed daily, also poses a risk to people’s health. However, whether *Cronobacter* infection in adults is due to contaminated food consumption is unproven [46]. The remaining 10 strains were from seven sequence types, reflecting their high diversity. Overall, these results indicate that imported dairy products on the Shanghai market were occasionally contaminated with *C. sakazakii* between 2005 and 2006.

PCR-probe based serotyping of *Cronobacter* spp. has been used to define 15 serotypes, of which 5 occur in *C. sakazakii*. Many of the serotypes are common across different species and, some were originally assigned to the wrong species due to strain mis-identification. This is therefore less discriminatory than 7-loci MLST with 645 variants to date. Ogrodzki & Forsythe (2015) proposed an alternative more reliable and discriminatory approach by in silico analysis of the *galF* and *gnd* genes which flank the O-antigen operon [34]. Table 1 shows the variation on O-serotype for the strains. Of particular interest was that all ST13 strains corresponded to Csak O: 2. Similarly, the ST13 strains from the 1994 NICU outbreak in France were serotype Csak O: 2 [34].

Since *Cronobacter* is a highly clonal genus, conventional genotyping methods are limited as they cannot always distinguish unrelated strains [1, 20]. This occurs with the application of PFGE and MLST with *Salmonella* and *Cronobacter*. This limitation can cause confusion and is one of the reasons for PulseNet changing to the application of WGS-SNP analysis [21, 22]. In this study, the *C. sakazakii* ST13 strains had been isolated from 6 countries and different dairy products. They were therefore likely to be unrelated, and so WGS-SNP analysis was undertaken to distinguish between these strains within the same sequence type.

**Fig. 2:**
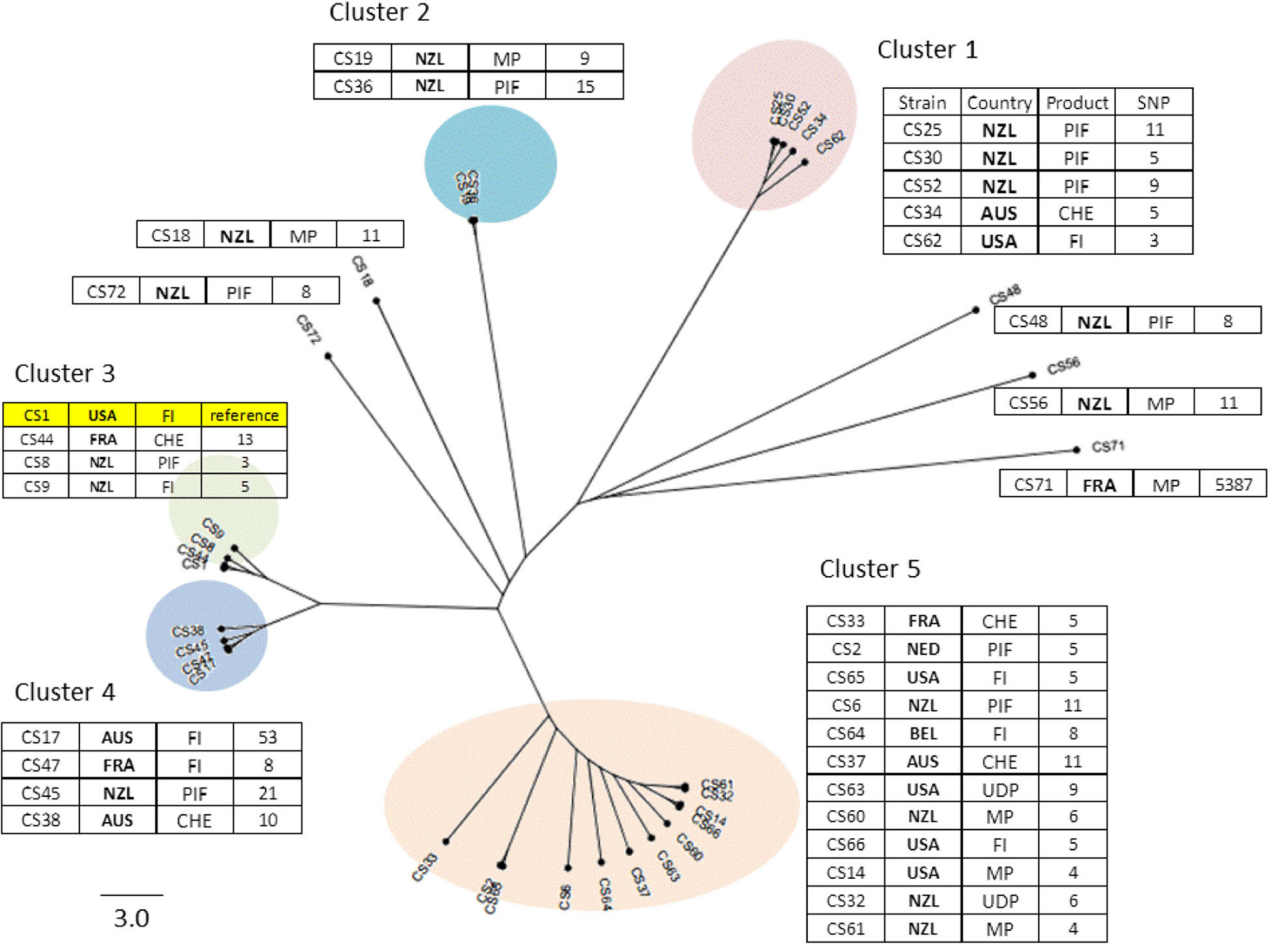
SNP phylogeny of C. sakazakii strains belonged to ST13. The SNPs werecalled using SMALT and SAMtools to generate the VCF files which were filtered usingVCFTools to include only SNPs with minimum quality score of 30, minimum depth of 8,and minimum allele frequency of 0.90. The SNPs in the cluster were concatenated andused to create a maximum likelihood phylogeny. The tree was rooted to midpoint. C.sakazakii CS-1 was the reference strain. The scale bar indicates the rate of nucleotidedifferences per sequence site

Figure 2 shows the SNPs tree analysis for the ST13 isolates with comparison to ST13 strain CS-1. The strains divided into 5 clusters based on the analytic parameters of times, locations and whole-genome SNPs. Thus, confirming that strains within the same ST are distinguishable. The small number of SNP differences was unexpected but may reflect the high clonality of the organism, which is also shown in the small variation reported for CRISPR-*cas* array profiles for strains within an ST which differ in their geographical and temperal sources [47, 48].

## Conclusion

The previous method, based on phenotyping, for the detection and identification of *E. sakazakii* from imported dairy products had resulted in false positive results for other Enterobacteriaceae; *E. coli, E. cloacae* and *F. helveticus*. The application of DNA-sequence based methods, such as 7-loci MLST, provides a more reliable means of identifying and genotyping presumptive *Cronobacter* isolates. The majority of formerly identified *E. sakazakii* isolates from imported dairy products were *C. sakazakii,* and included the pathovars ST4 and ST1. In this study the predominant sequence type was ST13 from dairy products imported from New Zealand. Due to the highly degree of clonality in the *Cronobacter* genus 7-loci MLST is unable to distinguish unrelated strains of *C. sakazakii.* Therefore, combining WGS-SNP with isolation date and geolocation is a better method for genotyping *C. sakazakii,* as it is traceable and allows the data to be shared worldwide. This approach considerably improves the success rate of food-borne pathogen traceability and surveillance, and subsequently are important for the protection of public health. In addition, WGS data can be used for studies beyond outbreak investigations, such as the detection of antibiotic resistance, virulence and thermotolerance genes.

## Additional file

Additional file 1: **Figure S1.** Neighbour-joining phylogenetic tree derived from *fus*A gene sequences (438 bp) for all isolates. (DOC 27 kb)

## Abbreviations

BIGSdb: Bacterial Isolate Genome Sequence database
CTAB: cetyl trimethyl ammonium bromide
FDA: Food and drug administration
ICMSF: the International Commission on Microbiological Specifications for Foods
MLST: Multi-locus sequence typing
MPS: Massively parallel sequencing
ST: Sequence type
STEC: Shiga toxin-producing *E. coli*
TSA: Tryptic soy agar L
VCF: variant call format
VRE: Vancomycin-resistant enterococci
WGS-SNP: Whole genome sequence with single nucleotide polymorphism analysis

## References

[1] Forsythe S (2018). Updates on the *Cronobacter* genus. Annu Rev Food Sci Technol.

[2] Joseph S, Cetinkaya E, Drahovska H, Levican A, Figueras MJ, Forsythe SJ (2012). *Cronobacter condimenti* sp. nov., isolated from spiced meat, and *Cronobacter universalis* sp. nov., a species designation for *Cronobacter* sp. genomospecies 1, recovered from a leg infection, water and food ingredients. Int J Syst Evol Microbiol.

[3] Joseph S, Sonbol H, Hariri S, Desai P, McClelland M, Forsythe SJ (2012). Diversity of the *Cronobacter* genus as revealed by multilocus sequence typing. J Clin Microbiol.

[4] Holy O, Forsythe S (2014). *Cronobacter* spp. as emerging causes of healthcare-associated infection. J Hosp Infect.

[5] Forsythe SJ, Dickins B, Jolley KA (2014). *Cronobacter*, the emergent bacterial pathogen *Enterobacter sakazakii* comes of age; MLST and whole genome sequence analysis. BMC Genomics.

[6] Caubilla-Barron J, Hurrell E, Townsend S, Cheetham P, Loc-Carrillo C, Fayet O (2007). Genotypic and phenotypic analysis of *Enterobacter sakazakii* strains from an outbreak resulting in fatalities in a neonatal intensive care unit in France. J Clin Microbiol.

[7] van Acker J, de Smet F, Muyldermans G, Bougatef A, Naessens A, Lauwers S (2001). Outbreak of necrotizing enterocolitis associated with *Enterobacter sakazakii* in powdered milk formula. J Clin Microbiol.

[8] Minor T, Lasher A, Klontz K, Brown B, Nardinelli C, Zorn D (2015). The per case and total annual costs of foodborne illness in the United States. Risk Anal.

[9] Friedemann M (2007). *Enterobacter sakazakii* in food and beverages (other than infant formula and milk powder). Int J Food Microbiol.

[10] Siqueira Santos RF, da Silva N, Amstalden Junqueira VC, Kajsik M, Forsythe S, Pereira JL (2013). Screening for *Cronobacter* species in powdered and reconstituted infant formulas and from equipment used in formula preparation in maternity hospitals. Ann Nutr Metab.

[11] Shaker R, Osaili T, Al-Omary W, Jaradat Z, Al-Zuby M (2007). Isolation of *Enterobacter sakazakii* and other *Enterobacter* sp. from food and food production environments. Food Control.

[12] Turcovský I, Kunikova K, Drahovska H, Kaclikova E (2011). Biochemical and molecular characterization of *Cronobacter* spp. (formerly *Enterobacter sakazakii*) isolated from foods. Antonie Van Leeuwenhoek.

[13] Caubilla-Barron J, Forsythe S (2007). Dry stress and survival time of *Enterobacter sakazakii* and other Enterobacteriaceae. J Food Protect.

[14] International Commission on Microbiological Specifications for Foods(ICMSF) (2002). Microorganisms in food 7: microbiological testing in food safety management. Accessed.

[15] Kluwer Academic/Plenum Publishers. Microorganisms in Food 7. In: Microbiological testing in food safety management. International Commission on Microbiological Specifications for Foods(ICMSF). 2002. https://link.springer.com/book/10.1007%2F978-1-4684-8369-7. Accessed 15 Jan 1999.

[16] Muytjens HLZH, Sonderkamp HJ, Kollée LA, Wachsmuth IK, Farmer JJ (1983). Analysis of eight cases of neonatal meningitis and Sepsis due to *Enterobacter sakazakii*. J Clin Microbiol.

[17] Himelright I, Harris E, Lorch V, Anderson M, Jones T, Craig A (2002). From the Centers for Disease Control and Prevention. J Am Med Assoc.

[18] Masood N, Moore K, Farbos A, Paszkiewicz K, Dickins B, McNally A (2015). Genomic dissection of the 1994 *Cronobacter sakazakii* outbreak in a French neonatal intensive care unit. BMC Genomics.

[19] Stranden A, Frei R, Widmer AF (2003). Molecular typing of methicillin-resistant *Staphylococcus aureus*: can PCR replace pulsed-field gel electrophoresis?. J Clin Microbiol.

[20] Alsonosi A, Hariri S, Kajsik M, Orieskova M, Hanulik V, Roderova M, Petrzelova J, Kollarova H, Drahovska H, Forsythe S, Holy O (2015). The speciation and genotyping of *Cronobacter* isolates from hospitalised patients. Euro J Clin Microbiol.

[21] Nadon C, Walle IV, Gernersmidt P. PulseNet International: Vision for the implementation of whole genome sequencing (WGS) for global food-borne disease surveillance. Euro Surveill. 2017;22(23). 10.2807/1560-7917.ES.2017.22.23.30544 PMC5479977 28662764

[22] Scharff RL, Besser J, Sharp DJ, Poe A, Zhang P, Concepción-Acevedo J, et al. An Economic Evaluation of PulseNet: A Network for Foodborne Disease Surveillance. Am J Prev Med. 2016; 50(5):S66–73. 10.1016/j.amepre.2015.09.018 26993535

[23] Kozyreva VK, Crandall J, Sabol A, Poe A, Zhang P, Concepción-Acevedo J, et al. Laboratory Investigation of Salmonella entericaserovar Poona Outbreak in California: Comparison of Pulsed-Field GelElectrophoresis (PFGE) and Whole Genome Sequencing (WGS) Results. PLoS Curr. 2016; 8:97–8. 10.1371/currents.outbreaks.1bb3e36e74bd5779bc43ac3a8dae52e6 PMC5145817 28018748

[24] Lytsy B, Engstrand L, Gustafsson A, Kaden R (2017). Time to review the gold standard for genotyping vancomycin-resistant enterococci in epidemiology: comparing whole-genome sequencing with PFGE and MLST in three suspected outbreaks in Sweden during 2013-2015. Infect Genet Evol.

[25] McRobb E, Sarovich DS, Price EP, Kaestli M, Mayo M, Keim P (2015). Tracing melioidosis back to the source: using whole-genome sequencing to investigate an outbreak originating from a contaminated domestic water supply. J Clin Microbiol.

[26] Ferdous M, Friedrich AW, Grundmann H, de Boer RF, Croughs PD, Islam MA (2016). Molecular characterization and phylogeny of Shiga toxin-producing *Escherichia coli* isolates obtained from two Dutch regions using whole genome sequencing. Clin Microbiol Infect.

[27] US Food and Drug Administration Center for Food Safety and Applied Nutrition (2002). Isolation and enumeration of *Enterobacter sakazakii* from dehydrated powdered infant formula.

[28] Microbial detection of *Enterobacter sakazakii*: Food and clinical. https://www.fda.gov/food/foodscienceresearch/laboratorymethods/ucm289378.htm.

[29] Kandhai MC, Reij MW, vall Puyvelde K (2004). A new protocol for the detection of *Enterobacter sakazakii* applied to environmental samples. J Food Prot.

[30] Government of Canada Laboratory Procedure. MFUP-27. The Dupont oualicon BAX system method for the detection of *Enterobacter sakazakii* in selected foods. Health products and food branch ATTA WA. Accessed Sep 2003.

[31] Li R, Zhu H, Ruan J, Qian W, Fang X, Shi Z (2010). *De novo* assembly of human genomes with massively parallel short read sequencing. Genome Res.

[32] Li R, Li Y, Kristiansen K, Wang J (2008). SOAP: short oligonucleotide alignment program. Bioinformatics.

[33] Joseph S, Desai P, Ji Y, Cummings CA, Shih R, Degoricija L (2012). Comparative analysis of genome sequences covering the seven *Cronobacter* species. PLoS One.

[34] Ogrodzki P, Forsythe S (2015). Capsular profiling of the *Cronobacter* genus and the association of specific *Cronobacter sakazakii* and *C. malonaticus* capsule types with neonatal meningitis and necrotizing enterocolitis. BMC Genomics.

[35] Li H, Handsaker B, Wysoker A, Fennell T, Ruan J, Homer N (2009). The sequence alignment/map format and SAMtools. Bioinformatics.

[36] Lorenc MT, Hayashi S, Stiller J, Lee H, Manoli S, Ruperao P (2012). Discovery of single nucleotide polymorphisms in complex genomes using SGSautoSNP. Biology.

[37] Sahl JW, Steinsland H, Redman JC, Angiuoli SV, Nataro JP, Sommerfelt H (2011). A comparative genomic analysis of diverse clonal types of enterotoxigenic *Escherichia coli* reveals pathovar-specific conservation. Infect Immun.

[38] Clark G, Paszkiewicz K, Hale J, Weston V, Constantinidou C, Penn C (2012). Genomic analysis uncovers a phenotypically diverse but genetically homogeneous *Escherichia coli* ST131 clone circulating in unrelated urinary tract infections. J Antimicrob Chemother.

[39] McNally A, Alhashash F, Collins M, Alqasim A, Paszckiewicz K, Weston V (2013). Genomic analysis of extra-intestinal pathogenic *Escherichia coli* urosepsis. Clin Microbiol Infect.

[40] Stamatakis A, Ludwig T, Meier H (2005). RAxML-III: a fast program for maximumlikelihood-based inference of large phylogenetic trees. Bioinformatics.

[41] Jackson EE, Forsythe SJ (2016). Comparative study of *Cronobacter* identification according to phenotyping methods. BMC Microbiol.

[42] Jarvis C (2005). New investigator award, blue ribbon abstract award. Am J Infect Control.

[43] Cui JH, Du XL, Wei RJ, Zhou HJ, Li W, Forsythe S, Cui ZG (2015). Multilocus sequence typing analysis of *Cronobacter* spp. isolated from China. Arch Microbiol.

[44] Joseph S, Forsythe SJ (2011). Predominance of *Cronobacter sakazakii* sequence type 4 in neonatal infections. Emerg Infect Dis.

[45] Hariri S, Joseph S, Forsythe SJ (2013). *Cronobacter sakazakii* ST4 strains and neonatal meningitis, United States. Emerg Infect Dis.

[46] Brandao MLL, Umeda NS, Jackson E, Forsythe SJ, de Filippis I (2017). Isolation, molecular and phenotypic characterization, and antibiotic susceptibility of *Cronobacter* spp. from Brazilian retail foods. Food Microbiol.

[47] Ogrodzki P, Forsythe SJ (2016). Clustered regularly interspaced short palindromic repeats (CRISPRs)-*cas* loci profiling of *Cronobacter sakazakii* pathovars. Future Microbiol.

[48] Ogrodzki P, Forsythe SJ (2017). DNA-sequence based typing of the *Cronobacter* genus using MLST, CRISPR-*cas* array and capsular profiling. Front Microbiol.

[49] Jolley KA, Maiden MC (2010). BIGSdb: scalable analysis of bacterial genome variation at the population level. BMC Bioinform.

