# Supplementary material for: DNA sequence-based re-assessment of archived Cronobacter sakazakii strains isolated from dairy products imported into China between 2005 and 2006
Source: BMC Genomics. 2018 Jun 28;19:506. doi: 10.1186/s12864-018-4881-9 (PMC6025729; doi:10.1186/s12864-018-4881-9)
Supplement: Supplementary file 1 — Figure S1. Neighbour-joining phylogenetic tree derived from fusA gene sequences (438 bp) for all isolates. (DOC 27 kb) [file 12864_2018_4881_MOESM1_ESM.doc]

Figure S1 Neighbour-joining phylogenetic tree derived from *fus*A gene sequences (438bp), showing positions of all *C. sakazakii* strains, CS-22 (*E. coli*), CS-23 (*F. helveticus*), and CS-35 (*E. cloacae*) isolates. Bootstrap values based on 1000 replications are shown at branch nodes. Bar, 1 substitution per 100 nucleotide positions.
